# Gait parameters and daily physical activity for distinguishing pre-frail, frail, and non-frail older adults: A scoping review

**DOI:** 10.1016/j.jnha.2025.100580

**Published:** 2025-05-14

**Authors:** Xin Zhang, Feng Li, Hans SM Hobbelen, Barbara C van Munster, Claudine JC Lamoth

**Affiliations:** a University of Groningen, University Medical Center Groningen, Department of Human Movement Sciences, 9713AV Groningen, the Netherlands; b Jilin University, School of Nursing, 965 Xinjiang Street, Changchun, China; c Hanze University of Applied Sciences, Research Group Healthy Ageing, Allied Health Care and Nursing, Groningen, the Netherlands; d University of Groningen, University Medical Center Groningen, Department of General Practice and Elderly Care Medicine, Groningen, the Netherlands; e University of Groningen, University Medical Center Groningen, University of Internal Medicine, Division of Geriatric Medicine, Groningen, the Netherlands

**Keywords:** Frailty screening, Gait parameters, Daily physical activity, Inertial sensors, Frailty phenotype

## Abstract

**Objective:**

This scoping review aimed to gather current knowledge on accurately identifying and distinguishing between non-frail, pre-frail, and frail older adults using gait and daily physical activity (DPA) parameters and/or models that combine gait with DPA parameters in both controlled and daily life environments.

**Methods:**

Following PRISMA-ScR guidelines, a systematic search was conducted across seven databases using key terms: "frail", "gait or walk", "IMU", and "age". Studies were included if they focused on gait analysis using Inertial Measurement Units (IMUs) for walking distances greater than 10 meters. Extracted data included study design, gait and DPA outcomes, walking conditions, and classification model performance. Gait parameters were grouped into four domains: spatio-temporal, frequency, amplitude, and dynamic gait. DPA parameters were synthesized into three categories: postural and transition, variability, and physical activity pattern.

**Results:**

A total of 15 cross-sectional studies involving 2,366 participants met the inclusion criteria. Gait analysis showed (pre)frail individuals had slower, shorter steps with longer stride times compared to non-frail individuals. Pre-frail individuals showed distinct gait patterns in periodicity, magnitude range, and variability. In daily activities, (pre)frail individuals displayed shorter, fragmented walking periods and longer transitions between positions. Walking variation identified pre-frail status, showing progressive decreases from non-frail to frail states. Combined gait and daily physical activity models achieved over 97% accuracy, sensitivity and specificity in distinguishing between groups.

**Discussion:**

This review provides an updated synthesis of the relationship between various gait and/or DPA parameters and physical frailty, highlighting gaps in pre-frailty detection and the variability in measurement protocols. It underscores the potential of long-term, sensor-based monitoring of daily physical activity for advancing pre-frailty screening and guiding future clinical trials.

Structured Abstract

**Background:**

Changes in gait and physical activity are critical indicators of frailty. With advancements in wearable sensor technology, long-term gait analysis using acceleration data has become more feasible. However, the contribution of parameters beyond gait speed, such as gait dynamics and daily physical activity (DPA), in identifying frail and pre-frail individuals remains unclear.

**Objective:**

This scoping review aimed to gather knowledge on accurately identifying and differentiating physical pre-frail and frail individuals from non-frail individuals using gait parameters alone or models that combine gait and DPA parameters, both in controlled settings and daily life environments.

**Methods:**

The review followed PRISMA-ScR guidelines. A search strategy incorporating key terms—"frail", "gait or walk", "IMU", and "age"—was applied across seven databases from inception to March 1, 2024. Studies were included if they focused on gait analysis in controlled or daily environments using Inertial Measurement Units (IMUs) and involved walking distances longer than 10 meters. Data on walking conditions, gait outcomes, classification methods, and results were extracted. Gait parameters were categorized into four domains: spatio-temporal, frequency, amplitude, and dynamic gait. DPA parameters were synthesized into three categories: postural and transition, variability, physical activity pattern.

**Results:**

A total of 15 cross-sectional observational studies met the eligibility criteria, covering 2,366 participants, with females representing 27%–80% of the sample and ages ranging from 60 to 92 years. Regarding gait parameters, (pre)frail individuals exhibited longer stride times, slower walking speeds, shorter steps, and reduced cadence compared to non-frail individuals. In three studies, pre-frail could be distinguished from the non-frail and frail group through gait periodicity, range of magnitude, and gait variability. DPA patterns differed between groups, with (pre)frail individuals showing shorter and more fragmented walking periods, brief walking bouts and longer postural transitions. Walking bout variation (CoV) effectively identified pre-frail status, decreasing 53.73% from non-frail to pre-frail, and another 30.87% from pre-frail to frail. Models combining both gait and DPA parameters achieved the highest accuracy (97.25%), sensitivity (98.25%), and specificity (98.25%) in distinguishing between groups.

**Discussion:**

This scoping review provides an updated overview of the current knowledge and gaps in understanding the relationship between gait parameters across different domains and DPA parameters along with physical frailty. Significant variability in gait measurement methods and protocols complicates direct comparisons between studies. The review emphasizes the need for further research, particularly in pre-frailty screening, and underscores the potential of inertial sensor-based long-term monitoring of daily physical activity for future clinical trials.

## Highlights

• IMU-based acceleration data from gait and daily activity patterns can identify differences between frail and non-frail older adults.
• There is a research gap in studies focusing on pre-frail status.
• Frailty classification accuracy improves when combining gait and multiple DPA parameters.
• Continuous monitoring of gait and physical activity through wearable sensors enables effective screening for pre-frailty in older adults.

## 1 Introduction

As the global population ages, the health burden related to age-associated functional decline and adverse events has increased significantly [1]. Frailty is often considered an extreme consequence of aging, characterized by increased vulnerability to external stressors [2]. It is associated with various negative health outcomes, including increased hospital admissions, readmissions, greater healthcare utilization, and rising medical costs [3]. One of the most widely used criteria for assessing physical frailty is the Fried Phenotype, which evaluates five components: low physical activity, slow walking speed, reduced grip strength, unintentional weight loss, and fatigue. The presence of one or more of these factors indicates physical frailty or pre-frailty [4].

The transition from healthy aging to frailty is a dynamic process. During this transition, the frailty symptoms can fluctuate, especially in the pre-frail stage. It is during this earlier phase that interventions may be most effective in preventing the progression to frailty [5]. Once frailty leads to incapacitation and disability, reversing the condition becomes increasingly challenging [2]. Therefore, the early identification of pre-frailty is crucial for delaying the aging process and preventing substantial disability and a decline in quality of life.

Changes in gait and physical activity patterns may serve as objective indicators of pre-frailty and frailty. In particular, slow gait speed has been recognized as an indicator of physical frailty [6]. Other Spatio-temporal gait parameters, such as stride time, step length, and cadence, have also been reported as sensitive measures for identifying pre-frail and frail older adults [7,8]. Clinical studies often assess gait in older populations using short walking distances (∼2.4 meters) with varying protocols [9,10], leading to inconsistent outcomes. Reliable gait speed measurement requires excluding the first and last 2.5 meters of the walking path to account for acceleration and deceleration, Furthermore, after achieving steady walking, reliable assessment requires a minimum of 4 gait cycles for Spatio-temporal parameters and 20 cycles for dynamic parameters [[11], [12], [13]].

In recent years, advancements in wearable sensor technology have made gait analysis of longer duration for clinical purposes accessible. These sensors enable data collection over extended walking durations, both in controlled laboratory settings and during daily living activities. From this data, a comprehensive array of complementary gait parameters can be derived to classify different age groups, assess fall risk, and evaluate frailty [[14], [15], [16]]. Furthermore, continuous monitoring in daily life settings allows for the extraction of daily physical activity (DPA) patterns, another hallmark of frailty.

Averaged gait parameters and moderate-to-vigorous physical activity (MVPA) derived from data collected using Inertial Measurement Units (IMUs) have been shown to correlate significantly with frailty status. Self-selected gait speed has been identified as the most relevant gait parameter for assessing frailty [[17], [18], [19]]. However, most studies have focused on short walking bouts (less than 10 meters), which do not adequately reflect an individual's functional capacity in daily life [10,20]. Additionally, the roles of parameters other than gait speed in identifying pre-frail and frail older adults, such as gait dynamics and DPA parameters, remain unclear. These parameters may offer greater sensitivity in distinguishing between various patient groups or levels of frailty [15,21,22]. Consequently, we reviewed studies that examined gait parameters derived from IMUs, encompassing different gait domains (e.g., Spatio-temporal and gait dynamics), with or without measures of physical activity. Our focus was specifically on walking assessments longer than 10 meters to determine which gait parameters effectively identify and differentiate pre-frailty and frailty from non-frail individuals in both controlled laboratory and daily life settings. The primary goal is to identify parameters derived from IMUs that can differentiate between frailty phenotypes for screening purposes. These parameters span different gait domains (including spatio-temporal and gait dynamics) in both controlled and daily life environments, along with measures of physical activity.

## 2 Methods

This scoping review was conducted in accordance with the JBI (Joanna Briggs Institute) methodology [23] for scoping reviews and drafted following the PRISMA-ScR (Preferred Reporting Items for Systematic Reviews and Meta-Analyses extension for Scoping Reviews) guidelines [24] (Supplement 3).

### 2.1 Eligibility criteria

Studies were included if their primary objective was to identify physical frailty or pre-frailty in older adults through gait analysis. Inclusion criteria comprised: studies involving adults aged ≥60 years (mean or median age), reporting associations between physical frailty and gait factors, gait parameters derived from inertial sensor signals, a walking distance of more than 10 meters, which we refer to as prolonged walking, and availability of full text.

The term (pre)frailty was used for studies that included both pre-frail and frail individuals without analyzing these groups separately. Exclusion criteria were conference proceedings, protocols, editorials, commentaries, reviews, correspondence, studies focused on specific diseases, case studies, or those centered on system/model development with fewer than 10 participants (Supplement 1-S1).

### 2.2 Search strategy and information sources

The search strategy was developed collaboratively by two authors. Seven databases—MEDLINE, Scopus, Cochrane, Web of Science, EBSCOhost, Embase, and IEEE Xplore—were searched on February 29, 2024. The search strategy included combinations of keywords and indexed terms: (Frail*’) AND (Gait*’ OR ‘Walk*’) AND (IMU*’) AND (Age*’). Full search criteria for each database are detailed in Supplement 1-S2. All retrieved records were uploaded to the online platform Rayyan [25], where duplicates were removed. Two independent authors screened titles and abstracts in parallel, and full texts were reviewed for eligibility. Discrepancies were resolved through discussion, with a third reviewer providing arbitration if necessary.

### 2.3 Quality assessment

The quality of the evidence was independently evaluated by three reviewers using a modified version of the Downs and Black checklist (Supplement 1-Table S1) [26,27].

### 2.4 Data extraction

The following data were extracted from the included studies: (1) General information (country, author, year, participant groups, age, gender distribution); (2) Walking test details (test environment, walking distance); (3) Sensor information (type, frequency, number, and placement of sensors); (4) Frailty definition; (5) Data processing descriptions; (6) Study outcomes; (7) Results related to differences between frailty categories and classification model performance (Table 1, Table 2, Table 3).

**Table 1:** Characteristics of included studies (n = 15).

Number, author, year, country	Group: sample size(n)	Age; % female	Frailty assessment instrument	Criteria of groups	Walking test		Sensors (Type, Frequency, Location)	Data Pre-processing (filter (low pass), window size (WS), size of WS(n))
[28] Álvarez-Millán et al., 2023, Mexico	non-frail: n = 15 frail: n = 31	>60; 27% non-frail:72.7 ± 2.5; 33% frail: 78.5 ± 1; 71%	FRAIL scale and Clinical Frailty Scale (CFS)	non-frail: didn't meet the criteria in either of the scales frail: meet both 2 scales: score of FRAIL > 2 and CFS>4	160 m walking test (A flat rectangular trajectory)		3D-accelerometer 100 Hz; chest	Calculated according to the output result of the system: Zephyr Bioharness 3.0
[29] Zhong et al., 2018, China	non-frail: n = 34 (pre)frail: n = 16	69.86 ± 9.27; 50% non-frail: 70.5 ± 8.6; 50% (pre)frail: 68.5 ± 10.7; 50%	FRAIL scale	non-frail: no phenotype (pre)frail: presenting 1 or more of five components	12 m walking test (only use 10 m analysis)		3D-accelerometer 100 Hz; lower back	filter: 10 Hz WS: *NA* n = 300
[30] Minici et al., 2022, Italy	non-frail: n = 11 (pre)frail: n = 23	>70; 38%	Fried Phenotype	non-frail: score 0 (pre)frail: score >0	60 m walking test (20 m long path)		3D-accelerometers 102.4 Hz; wrist, lower back	filter: 20 Hz WS: 9 gait segments; n = 34
[31] Fan et al., 2023, China	non-frail: n = 186 frail: n=28	68.9 ± 6.7; 73% non-frail: 68.1 ± 6.1; 75% frail: 74.3 ± 8.4; 61%	Fried Phenotype	non-frail: score 0−2 frail: 3−5	6-min walk test (6 MWT)		*NA;* thigh	Filter: *NA* WS: *NA* n = 214
[32] Abbas et al., 2022, France	non-frail: n = 16 pre-frail: n = 18 frail: n = 16	70−92; *NA*	Fried Phenotype	non-frail: score 0 pre-frail: score 1−2 frail: 3−5	free-living		3D-accelemeters 50 Hz; body trunk 25 Hz; chest	filter: 25 Hz WS: 6 s n = 150
[33] Schmidle et al., 2023, Germany and France	non-frail: n = 12 pre-frail: n = 33 frail: n = 43	80.6 ± 9.1; 55% non-frail: 72.5 ± 5.8; 33% pre-frail: 80.9 ± 9.3; 45% frail: 82.7 ± 8.6; 67%	Questionnaire based on the Fried Phenotype	non-frail: score 0 pre-frail: score 1−2 frail: 3−5	free-living (at least 6days/21days, at least 8 h between 8am-8pm)		Huawei 2 (4 G) smartwatch	Filter: *NA* WS: 5 s n = 88
[34] Kumar et al., 2021, USA	non-frail: n = 29 (pre)frail: n = 34	> = 65; 80% non-frail: 74.97 ± 7.10; 89% (pre)frail: 81.26 ± 8.94; 73%	Fried Phenotype	non-frail: score 0 (pre)frail: score>0	free-living (2 consecutive days)		3D-accelerometer 50 Hz; chest	filter: 2.5 Hz WS: 60 s n = 63
[35] Kumar et al., 2023, USA	non-frail: n = 44 pre-frail: n = 60 frail: n**=**22	> = 65; 79% non-frail: 74.6 ± 6.5; 84% pre-frail: 79.72 ± 8.6; 75% frail: 82.81 ± 9.79; 86%	Fried Phenotype	non-frail: score 0 pre-frail: score 1−2 frail: 3−5	free-living (2 consecutive weekdays)		3D-accelerometer 50 Hz; chest	Filter: *NA* WS: > = 20 s n = 116
[36] Abbas et al., 2023, France	non-frail: n = 9 frail: n = 9	80−92; *NA*	Fallen history and Fried Phenotype	non-frail: score 0 according to Fried Phenotype frail: fallen at least once in the last year	free-living (24 h)	3D-accelerometer 50 Hz; body trunk		Filter: *NA* WS: 30 s n = 570
[37] Park et al., 2021, USA	non-frail: n = 73 (pre)frail: n = 186	76.0 ± 9.8; 65% non-frail: 74.4 ± 6.6; 73% (pre)frail: 76.6 ± 8.4; 62%	Fried Phenotype	non-frail: score 0 (pre)frail: score>0	free-living (2 consecutive weekdays)	3D-accelerometer 50 Hz; body trunk		system-based：PAMWare™ (BioSensics, USA)
[38] Jansen et al., 2019, Germany	non-frail: n = 40 pre-frail: n = 53 frail: n = 19	> = 65; 79% non-frail: 74.7 ± 1.6; 85% pre-frail: 80.5 ± 8.7; 74% frail: 82.8 ± 8.8; 90%	Fried Phenotype	non-frail: score 0 pre-frail: score 1−2 frail: 3−5	free-living (2 consecutive weekdays)	inertial sensors *NA;* shank, thighs, lower back		system-based：LEGSys™; BioSensics, Cambridge, USA
[39] Din et al., 2020, Tanzania	non-frail: n = 35 frail: n = 24	> = 60; 63% non-frail: 70.2 ± 8.41; 53% frail: 80.3 ± 12.38; 76%	Comprehensive Geriatric Assessment	diagnosed by geriatric doctors	free-living 1week	3D-accelerometer 100 Hz; fifth lumbar vertebra		Filter: *NA* WS: *NA* n = 65
[40] Camerlingo et al., 2023, USA	non-frail: n = 21 pre-frail: n = 23 frail: n = 6	72.26 ± 5.02; 52% non-frail: 71.10 ± 3.59; 52% pre-frail: 73.74 ± 5.52; 39% frail: 70.70 ± 6.53; 100%	Fried Phenotype	non-frail: score 0 pre-frail: score 1−2 frail: 3−5	free-living 2week	3D-accelerometer 100 Hz; fifth lumbar vertebra; wrist		Filter: *NA* WS: *>3 gait cycle* n = 49
[41] Kumar et al., 2020, Mexico	non-frail: n = 44 (pre)frail: n = 82	> = 65; 80% non-frail: 74.6 ± 6.5; 86% (pre)frail: 81.2 ± 8.6; 76%	Fried Phenotype	non-frail: score 0 (pre)frail: score>0	free-living (2 consecutive days)	3D-accelerometer 50 Hz; chest		filter: NA WS: 20 s/30 s/40 s/50 s/60 s/>60 s; n = 126 (60 s: n = 94)
[42] Soltani et al., 2021, Switzerland	frail & non-frail: n = 1071	>65; 35.48%	gender, BMI, and handgrip strength	frail: same as the cut-off value of Fried Phenotype non-frail: didn't meet criteria	free-living at least 8 valid days/13 days	3D-accelerometer 50 Hz; wrist		filter: NA WS: 30 s/30−120 s/>120 s n = 1071

**Table 2:** Comparison of gait parameters between non-frail and (pre)frail groups (n = 14).

Number, author, year, country	Gait and DPA parameters	Statistics	Comparison between groups (significant, effect size)	Description of parameter’s values: ((pre)frail vs non-frail (|(pre)frail - non-frail|, |(pre)frail - non-frail|*100%/non-frail)
***In Laboratory Assessment***				
[28] Álvarez-Millán et al., 2023, Mexico	**1. Spatio-temporal:** average of: ①walking speed (m/sec.), ②cadence (steps/min), ③step length (m), normalized ④cadence & ⑤step length (m); **2. Frequency domain:***NA***3. Amplitude domain:** Range of acceleration (g) at ⑥magnitude, ⑦AP, ⑧ML, ⑨VT； RMS of acceleration (g) at ⑩magnitude, ⑪AP, ⑫ML, ⑬VT **4. Gait dynamics:***NA***5.Others-Ratios of acceleration:** ⑭step length/cadence (m.s), ⑮normalized step length/cadence (m.s), ⑯range of aAP/aML, ⑰ normalized range of aAP/aML	**Comparison between groups:** One-way ANOVA **Effect size:** Cohen's d#	**1. Spatio-temporal:*p*<0.05** ①②③④⑤ **d>0.5** (① 5.62, ② 3.57, ③ 5.1, ④ 3.89, ⑤ 6.5) **3. Amplitude domain:*p*<0.05** ⑥⑦⑧⑨⑩⑪⑬ **d>0.5** (⑥ 4.71, ⑦ 4.80, ⑧ 4.64, ⑨ 5.70, ⑩ 4.24, ⑪ 5.66, ⑫ 5.06, ⑬ 3.33) **5. Others-Acceleration ratios:*p*<0.05** none **d>0.5** (⑭4.0, ⑮4.0, ⑯2.42, ⑰1.10)	**pre-frail/frail < non-frail** ① 0.74 vs 1.05 (0.31, 29.52%) ② 103.1 vs 114.35 (11.25, 9.84%) ③ 0.42 vs 0.55 (0.13, 23.64%) ④ 103.53 vs 114.05 (10.52, 9.22%) ⑤ 0.42 vs 0.55 (0.13, 23.64%) ⑥ 0.8 vs 1.06 (0.26, 24.53%) ⑦ 0.39 vs 0.56 (0.17, 30.36%) ⑧ 0.58 vs 0.79 (0.21, 26.58%) ⑨ 0.32 vs 0.41 (0.09, 21.95%) ⑩ 0.09 vs 0.12 (0.03, 25.00%) ⑪ 0.1 vs 0.14 (0.04, 28.57%) ⑬ 0.09 vs 0.1 (0.01, 10.00%) **pre-frail/frail > non-frail: none**
[29] Zhong et al., 2018, China	**1. Spatio-temporal:** ①walking speed (m/s) **2. Frequency domain**: ②step frequency (Hz) **3. Amplitude domain:** RMS of ③AP, ④ML, ⑤VT **4. Gait dynamics:** variability of ⑥AP, ⑦ML, ⑧VT; ⑨step variability, ⑩step regularity, and ⑪step symmetry	**Comparison between groups:** MONCOVA **Effect size:** partial eta square (ηp2)	**1. Spatio-temporal (**①**)*p*<0.05** (0.001); **ηp2 > 0.09** (0.232) **2. Frequency domain (**②**)*p*<0.05** none; **ηp2 > 0.09** none **3. Amplitude domain*p*<0.05** (③0.019, ④0.002, ⑤0.002) **ηp2 > 0.09** (③0.118, ④0.197, ⑤0.196) **4. Gait dynamics*p*<0.05** (⑧0.022, ⑩0.011) **ηp2 > 0.09** (⑧0.114, ⑩0.138)	**pre-frail < non-frail** ① 1.03 vs 1.24 (0.21, 16.94%) ③ 0.14 vs 0.16 (0.02, 12.50%) ④ 0.10 vs 0.13 (0.03, 23.08%) ⑤ 0.16 vs 0.22 (0.06, 27.27%) ⑧ 0.12 vs 0.15 (0.03, 20.00%) ⑩ 0.59 vs 0.68 (0.09, 13.24%) **pre-frail > non-frail: none**
[30] Minici et al., 2022, Italy	**1. Spatio-temporal:***NA***2. Frequency domain:** mean, median, standard deviation, minimum and maximum values, interquartile range (IQR), and Kurtosis, and Skewness of FFT coefficients **3. Amplitude domain:** acceleration at AP, VT, ML, and magnitude: mean, median, standard deviation, minimum and maximum values, interquartile range (IQR), mean absolute deviation (MAD), root mean square (RMS), kurtosis, zero-crossing rate (ZCR) mean, median, standard deviation, minimum and maximum values, interquartile range (IQR), and Kurtosis, and Skewness of CWT coefficient **4. Gait dynamics:***NA*	**Comparison between groups:** ANOVA **Effect size:** Cohen's d#	**3. Amplitude domain (**RMS on the acceleration magnitude**):*p*<0.05** (wrist & lower back <0.001) **d>0.5** (lower back 3.03) **4. Gait dynamics:***NA*	**pre-frail/frail < non-frail: RMS on the acceleration magnitude** lower back: 1.04 vs 1.06 (0.02, 1.89%) **frail > non-frail: *NA***
[31] Fan et al., 2023, China	**1. Spatio-temporal:** ①large step gait speed (m/s), ②average gait speed (m/s), ③total cadence (step/min), ④large step cadence (step/s), ⑤step length (m), ⑥step time (s) **2. Frequency domain:***NA***3. Amplitude domain**: *NA***4. Gait dynamics:***NA*	**Comparison between groups:** Independent samples t-tests **Effect size:** Cohen's d#	**1. Spatio-temporal*p*<0.05** (① <0.001, ② <0.001, ③0.023, ④0.003, ⑤ <0.001, ⑥0.004) **d>0.5** (①0.83, ②0.78, ③0.51, ④0.65, ⑤0.68, ⑥0.56)	**frail < non-frail** ① 0.96 vs 1.18 (0.22, 18.64%) ② 0.96 vs 1.17 (0.21, 17.95%) ③ 113.5 vs 120.4 (6.90, 5.73%) ④ 109.6 vs 118.9 (9.30, 7.82%) ⑤ 0.51 vs 0.59 (0.08, 13.56%) **frail > non-frail** ⑥ 0.53 vs 0.50 (0.03, 6.00%)
***In Daily Life Assessment***				
[32] Abbas et al., 2022, France	**1. Spatio-temporal:** ①average cadence in 6 sec windows **2. Frequency domain:** ②periodicity of the signal **3. Amplitude domain:** ③25% range of the acceleration magnitude **4. Gait dynamics:** ④variability-time series **5. Others:** ⑤⑥standard deviation and kurtosis of the outcome of AR model	**Comparison between groups:** 3 groups-Kruskal-Wallis H Test 2 groups Wilcoxon Rank Sum Test **Effect size:***NA*	**Among 3 groups*p*<0.05** (①②③④⑤⑥ <0.001) **Pairwise comparison between Non-frail, Pre-frail, and Frail*p*<0.05 1. Spatio-temporal** ① (P-F <0.001, N-F <0.01) **2. Frequency domain** ② (N-P & P-F & N-F <0.001) **3. Amplitude domain** ③ (N-P & P-F & N-F <0.001) **4. Gait dynamics** ④ (N-P & P-F & N-F <0.001) **5. Others** ⑤ (N-P & P-F & N-F <0.001) ⑥ (N-P <0.01, N-F <0.001)	***NA***
[33] Schmidle et al., 2023, Germany and France	**1. Spatio-temporal:** cadence (steps/min) **2. Frequency domain:***NA***3. Amplitude domain**: mean amplitude deviation **4. Gait dynamics:***NA*	**Association of parameters:** Spearman correlation **Effect size:***NA*	**1. Spatio-temporal** (cadence) ***p*<0.05** (<0.001, R^2^ = 0.25) **3. Amplitude domain** (mean of amplitude deviation) ***p*<0.05** (0.016, R^2^ = 0.07)	***NA***
[34] Kumar et al., 2021, USA	**Gait Parameters1. Spatio-temporal:** ①stride time (sec.), ②step time (sec.) **2. Frequency domain:** ③dominant frequency (Hz) **3. Amplitude domain:***NA***4. Gait dynamics:** ④PSD slope (W), ⑤variability-stride; ⑥variability-step; ⑦irregularity-time delay (ms); ⑧irregularity-sample entropy **5. Others-Gait repeatability of:** ①-⑧	***NA***	***NA***	**pre-frail/frail < non-frail** ③ 1.75 vs 1.93 (0.18, 9.33%) ④ 0.57 vs 1.59 (1.02, 64.16%) ⑤ 8.68 vs 9.19 (0.51, 5.55%) ⑥ 10.79 vs 11.01 (0.22, 2.00%) Repeatability 0.01−0.76 vs 0.13−0.87 (0.33, 57.89%) **pre-frail/frail > non-frail** ① 1.22 vs 1.10 (0.12, 10.91%) ② 0.61 vs 0.55 (0.06, 10.91%) ⑦ 162.28 vs 143.29 (18.99, 13.25%) ⑧ 0.99 vs 0.87 (0.12, 13.79%)
	**DPA Parameters1. Postural and Transition:** ①Total number of steps (24 h), ②total walking duration (min./24 h) **2. Variability***: NA***3. PA pattern:***NA***4. Others: Repeatability of:** ①②			**pre-frail/frail < non-frail** ① 1498.29 VS 3342.17 (1843.88, 55.17%) ② 29.42 VS 55.18 min (25.76, 46.68%) Repeatability 0.13&0.15 vs 0.29&0.25 (0.14, 51.85%) **pre-frail/frail > non-frail: *NA***

Number, author, year, country	Gait and DPA parameters	Statistics	Comparison between groups (significant, effect size)	Description of parameter’s values: ((pre)frail vs non-frail (|(pre)frail - non-frail|, |(pre)frail - non-frail|*100%/non-frail)
[35] Kumar et al., 2023, USA	**Gait Parameters1. Spatio-temporal:** ①stride time (sec.) **2. Frequency domain:***NA***3. Amplitude domain:***NA***4. Gait dynamics:** ②PSD slope(W)	**Comparison between groups:** univariate ANOVA **Effect size:** Cohen’s d	**Pairwise comparison between Non-frail, Pre-frail, and Frail 1. Spatio-temporal** (①) ***p*<0.05** (N-P 0.009, N-F 0.002) **d>0.5** (N-P 0.85, N-F 1.11) **2. Frequency domain** (②) ***p*<0.05** (N-P 0.037, N-F 0.036) **d>0.5** (N-P 0.80, N-F 1.02)	**Among 3 groups** ① 1.27 vs 1.23 vs 1.17 (N-P 0.06, 5.13%; P-F 0.04, 3.25%) ② 0.26 vs 0.38 vs 0.75 (N-P -0.37, 49.33%; P-F -0.12, -31.58%)
	**DPA Parameters1. Postural and Transition:** ①lying duration (sec./48 h), ②sitting duration (sec./48 h), ③standing duration (sec./48 h), ④walking duration (sec./48 h), duration of ⑤SiSt (sec./48 h), ⑥StSi (sec./48 h) **2. Variability**: Cov of ⑦lying (%), ⑧sitting (%), ⑨standing (%), ⑩walking (%), ⑪SiSt (%), ⑫StSi (%) **3. PA pattern:***NA*		**1. Postural and Transition*p*<0.05** ① (N-P 0.048) ⑥ (N-P 0.047, N-F 0.025) **d>0.5** ① (N-P 0.60, N-F 0.63) ② (N-F 0.73), ③ (P-F 0.54), ⑤ (N-F 0.71), ⑥ (N-P 0.59, N-F 0.82) **2.Variability**: ***p*<0.05** ⑦ (N-P 0.021, N-F 0.001) ⑩ (N-P 0.029, N-F 0.001, P-F 0.05) ⑫ (N-P 0.047, N-F 0.025) **d>0.5** ⑦ (N-F 1.11, P-F 0.52), ⑪ (N-F 0.76) ⑩ (N-P 0.66, N-F 1.32, P-F 0.80) ⑫ (N-P 0.55, N-F 0.74)	**Among 3 groups** ① 2693.63 vs 2714.28 vs 1955.33 (N-P 758.95, 38.81%; P-F -20.65, -0.76%) ⑥ 3.64 vs 3.46 vs 3.16 (N-P 0.3, 9.49%; P-F 0.18, 5.20%) ⑦ 129.20 vs 157.71 vs 188.70 (N-P -30.99, -16.42%) ⑩ 94.06 vs 136.06 vs 189.79 (N-P -53.73, -28.31%; P-F -42, -30.87%) ⑫ 28.6 vs 27.06 vs 23.73 (N-P 3.33,14.03%; P-F 1.54, 5.69%)
[36] Abbas et al., 2023, France	**Gait Parameters1. Spatio-temporal:***NA***2. Frequency domain:** ①periodicity **3. Amplitude domain:***NA***4. Gait dynamics:***NA*	**Comparison between groups:** Wilcoxon rank sum test **Effect size:** Cohen's d#	**1. Frequency domain (**①**)*p*<0.05** (<0.001); **d>0.5** (1.49)	**frail < non-frail: periodicity** 0.23 vs 3.5 (3.27, 93.43%)**frail > non-frail:***NA*
	**DPA Parameters1. Postural and Transition:** ①steps (24 h) **2. Variability**: *NA***3. PA pattern:** ②activity rate, ③energy expenditure (EE), ④sleep efficiency		**1. Postural and Transition** (①) ***p*<0.05** (<0.001); **d>0.5** (5.76)**3. PA pattern** (③)***p*<0.05** (0.001);**d>0.5** (2.3)	**frail < non-frail** ① 1019 vs 4663 (3644, 78.15%) ③ 19.7 vs 26.6 (6.9, 25.94%) **frail > non-frail: none**
[37] Park et al., 2021, USA	**Gait Parameters1. Spatio-temporal:** cadence (steps/min) **2. Frequency domain:***NA***3. Amplitude domain:***NA***4. Gait dynamics:***NA*	**Comparison between groups:** univariate ANOVA **Effect size:** Cohen’s d	**1. Spatio-temporal** (cadence) ***p*<0.05** (<0.0001); **d>0.5** (0.5)	**pre-frail/frail < non-frail: cadence** 108.8 vs 115.3 (6.5, 5.64%) **pre-frail/frail > non-frail: *NA***
	**DPA Parameters1. Postural and Transition:** ①% of lying, ②% of sitting, ③% of standing, ④% of walking, ⑤ steps (24 h), duration of (90th percentile) ⑥SiSt (sec.), ⑦StSi (sec.); number of ⑧SiSt, ⑨StSi **2. Variability**: *NA***3. PA pattern:** ⑩maximal steps in walking bouts, ⑪average steps of walking bouts,		**1. Postural and Transition：*p*<0.05** (②0.04, ③ <0.0001, ④ <0.0001, ⑤<0.0001, ⑥ <0.001, ⑦0.004, ⑧0.023, ⑨0.032)**d>0.5** (③ 0.81, ④ 0.82, ⑤ 0.69, ⑥ 0.61)**3. PA pattern:*p*<0.05 (**⑩ <0.0001, ⑪0.003) **d>0.5** (⑩0.68)	**pre-frail/frail < non-frail** ③ 13.7 vs 17.9 (4.2, 23.46%) ④ 4.3 vs 6.8 (2.5, 36.76%) ⑤ 3004.6 vs 4788.5 (1783.9, 37.25%) ⑩ 472.9 vs 1372.7 (899.8, 65.55%)**pre-frail/frail > non-frail** ⑥ 4.6 vs 3.9 (0.7, 17.95%)
[38] Jansen et al., 2019, Germany	**Gait Parameters1. Spatio-temporal:** normal walking speed (m/s)**2. Frequency domain:***NA***3. Amplitude domain:***NA***4. Gait dynamics:***NA*	**Comparison between groups:** One-way ANOVA**Effect size:** Cohen's d#	**1. Spatio-temporal** (normal walking speed)***p*<0.05** (<0.001)**d>0.5** (N-F 2.62, N-P 1.38, P-F 1.19)	**Among 3 groups: walking speed** 0.64 vs 0.92 vs 1.18 (N-P 0.26, 22.03%; P-F 0.14, 15.18%)
	**DPA Parameters1. Postural and Transition**: *NA***2. Variability:***NA***3. PA pattern:** ①average steps of walking bouts, ②maximal steps of walking bout, ③% active activity		**3. PA pattern:*p*<0.05** (①0.025, ②③ <0.001) **d>0.5** ① (N-P 0.63) ② (N-P 0.84, N-F 1.11, P-F 0.66) ③ (N-P 0.93, N-F 1.19)	**Among 3 groups** ① 27 vs 33 vs 39 (N-P -6, -15.38%; P-F -6, -18.18%) ② 285 vs 591 vs 1668 (N-P -1077, 64.57%; P-F -306, -51.78%) ③ 16.4 vs 18.9 vs 25.0 (N-P -6.1, -24.4%; P-F -2.5, -13.23%)
[39] Din et al., 2020, Tanzania	**Gait Parameters1. Spatio-temporal:***NA***2. Frequency domain:***NA***3. Amplitude domain**: *NA***4. Gait dynamics:** gait variability	**Comparison between groups:** independent sample t-tests **Effect size:** Cohen's d#	**4. Gait dynamics** (gait variability) ***p*<0.05** (0.023); **d>0.5** (0.738)	**frail < non-frail: gait variability** 0.43 vs 0.58 (0.15, 25.86%) **frail > non-frail: *NA***
	**DPA Parameters1. Postural and Transition:** ①duration of walking(min./24 h), ②% of walking, ③number of walking bouts (24 h), ④steps (24 h) **2. Variability:***NA***3. PA pattern:** ⑤average length of walking bout (sec.) (7days), ⑥proportion of shorter walking bouts (7days)		**1. Postural and Transition:*p*<0.05** (①②③④ <0.001) **d>0.5** (①1.74, ②2.52, ④1.72) **3. PA pattern**: ***p*<0.05** (⑤0.031, ⑥0.049) **d>0.5** none	**frail < non-frail** ① 14.04 vs 99.51 (85.47, 85.89%) ② 0.98 vs 6.91 (5.93, 85.82%) ④ 1029 vs 8409 (7380, 87.76%) **frail > non-frail: none**
[40] Camerlingo et al., 2023, USA	**Gait Parameters1. Spatio-temporal:** ①average gait speed (m/s), ②step time (s), ③step length (m), ④stance time (s), ⑤double support (s), ⑥initial double support (s), ⑦terminal double support (s), ⑧cadence (step/min) **2. Frequency domain:***NA***3. Amplitude domain**: *NA***4. Gait dynamics:***NA*	**Comparison between groups:** ANOVA and Tukey's HSD pairwise tests **Effect size:** Cohen's d#	**Between non-frail and (pre)frail 2 groups** (Spatio-temporal): ***p*<0.05** (①0.012, ②0.045, ④0.045, ⑤0.034, ⑥0.029, ⑦0.031) **d>0.5** (①0.76, ②0.57, ③0.50, ④0.662, ⑤1.00, ⑧0.60) **Pairwise comparison between Non-frail, Pre-frail, and Frail*p*<0.05** ① (N-P 0.042, N-F 0.028), ⑤ (N-F 0.047), ⑦ (N-F 0.045) **d>0.5** ① (N-P 0.64, N-F 1.64, P-F 0.82), ② (N-P 0.57, N-F 0.42), ③ (N-F 1.37, P-F 0.98), ④ (N-P 0.66), ⑤ (N-P 1.00), ⑧ (N-P 0.67)	**Between 2 groupspre-frail/frail < non-frail:** ① 0.83 vs 0.91 (0.08, 8.79%) **pre-frail/frail > non-frail:** ② 0.63 vs 0.61 (0.02, 3.28%) ④ 0.81 vs 0.78 (0.03, 3.85%) ⑤ 0.35 vs 0.33 (0.02, 6.06%) **Among the 3 groups** ① 0.77 vs 0.84 vs 0.91 (N-P 0.06, 6.59%; P-F 0.07, 8.33%) ⑤ 0.34 vs 0.35 vs 0.33 (N-P 0.02, 6.06%; P-F 0.01, 2.86%)
	**DPA Parameters1. Postural and Transition:** ①walking duration (min/24 h) **2. Variability:***NA***3. PA pattern:** ②sleep duration(hr/24 h), ③average bout duration (s), ④sedentary (hr/24 h), ⑤light PA (hr/24 h), ⑥moderate PA (hr/24 h), ⑦vigorous PA (hr/24 h) **4. Others:** ⑧mean of SVMg, ⑨5^th^ of SVMg, ⑩95^th^ of SVMg		**Between non-frail and (pre)frail 2 groups*p*<0.05** (⑥0.011, ⑧0.027, ⑩0.006) **d>0.5** (④ 0.51, ⑤ 0.42, ⑥ 0.87, ⑧ 0.77, ⑩ 0.94) **Pairwise comparison between Non-frail, Pre-frail, and Frail*p*<0.05** ②(N-F 0.007, P-F 0.016), ④(N-F 0.011, P-F 0.041), ⑥(N-P 0.018), ⑩(N-P 0.013); **d>0.5** ②(N-F 0.95, P-F 0.88), ④(N-P 0.40, N-F 0.90, P-F 0.47), ⑤(N-P 0.47), ⑥(N-P 0.90, N-F 0.77), ⑦(N-F 0.94, P-F 0.47), ⑧(N-P 0.85, N-F 0.55), ⑨(N-F 0.44), ⑩(N-P 0.96, N-F 0.84)	**Between 2 groupspre-frail/frail < non-frail** ⑥ 1.14 vs 1.86 (0.72, 38.71%) ⑧ 114 vs 132 (18, 13.64%) ⑩ 327 vs 398 (71, 17.84%) **pre-frail/frail > non-frail: noneAmong the 3 groups** ② 3.54 vs 5.02 vs 5.18 (N-P 0.16, 3.09%; P-F 1.48, 29.48%) ④ 13.0 vs 12.1 vs 11.4 (N-P 0.7, 6.14; P-F 0.9, 7.44%) ⑥ 1.27 vs 1.10 vs 1.86 (N-P -0.73, 39.25%; P-F 0.17, 15.45%) ⑩ 338 vs 324 vs 398 (N-P -74, 18.59%; P-F 14, 4.32%)
[41] Kumar et al., 2020, Mexico	**Gait Parameters1. Spatio-temporal:** ①step time (sec.), ②stride time (sec.) **2. Frequency domain:** ③PSD max (W/Hz), ④PSD width (Hz), ⑤Dominant frequency (Hz) **3. Amplitude domain:***NA***4. Gait dynamics:** ⑥PSD slope (W), ⑦variability-step, ⑧variability-stride, ⑨asymmetry, ⑩irregularity-time delay (ms), ⑪irregularity- sample entropy (bits)	**Comparison between groups:** univariate ANOVA **Effect size:** Cohen’s d	**1. Spatio-temporal*p*<0.05** (①② <0.001) **d>0.5** (①0.91, ②0.92) **2. Frequency domain*p*<0.05** (③⑤⑥ <0.001) **d>0.5** (③0.90, ⑤0.90, ⑥0.97)	**pre-frail/frail < non-frail** ③ 0.07 vs 0.17 (0.1, 58.82%) ⑤ 1.73 vs 1.90 (0.17, 8.95%) ⑥ 0.48 vs 1.24 (0.76, 61.29%) **pre-frail/frail > non-frail** ① 0.61 vs 0.56 (0.05, 8.93%) ② 1.23 vs 1.13 s (0.1, 8.85%)
	**DPA Parameters1. Postural and Transition:***NA***2. Variability**: ①CoV of walking bouts (%) **3. PA pattern:** ②number of continuous walk (48 h), ③total continuous walking duration (60 s, sec./48 h), ④max walking bouts (sec./48 h), ⑤max number of continuous steps (48 h), ⑥% of non-continuous walking duration (48 h)		**2. Variability** (②)***p*<0.05** (0.002); **d>0.5** (0.69) **3. PA pattern:*p*<0.05** (③④⑤ 0.001) **d>0.5** (③0.70, ④0.77, ⑤0.78)	**pre-frail/frail < non-frail** ② 195.55 vs 252.74 (57.19, 22.63) ③ 2436.79 vs 4042.33 s (1605.54, 39.72%) ④ 216.98 vs 475.62 s (258.64, 54.38%) ⑤ 896.63 vs 1867.58 (970.95, 51.99%) **pre-frail/frail > non-frail: none**

*NA*, no information was reported; AP, Anteroposterior; ML, mediolateral; VT, vertical; PSD, Power Spectral Density; RMS, Root Mean Square; PA, Daily Physical Activity; CoV, Coefficient of Variation; StSi, Stand to Sit; SiSt, Sit to Stand; MtV, Moderate to Vigorous Physical Activity; ANOVA, Analysis of Variance; MONCOVA, multivariate analysis of covariance; # Cohen’s d calculated manually by mean and standard deviation; N-P, non-frail vs pre-frail; N-F, non-frail vs frail; F-P, frail vs pre-frail; ML, Machine Learning; NN, Neural Network; SVM, Support Vector Machine; R, Regression; KNN, k-Nearest Neighbors; RF, Random Forest; GBM, Gradient Boosting Machine; NB: Naive Bayes; Log Logistic Regression; Acc., Accuracy; Sens., Sensitivity; Spec., Specificity; AUC, Area Under the Curve; AIC, Akaike Information Criterion; BIC, Bayesian Information Criterion; RFE, recursive feature elimination technique.

**Table 3:** Performance of frailty classification model (n = 7).

Number, author, year, country	Gait Parameters	DPA parameters	Statistics	Results
***Classification model based on gait parameters (n = 4)***				
[30] Minici et al., 2022, Italy	**Gait Features extracted (n = 125) 1. Spatio-temporal 2. Frequency domain 3. Amplitude domain 4. Gait dynamics:***NA*	***NA***	**Features selection:** descending order by ANOVA F-statistic **Features contribution**: sort by Acc. value **Classification:** 5 ML (NB; RF; Log, ML perceptron, SVM)	**1. Features selection:***NA***2. Features contribution:** amplitude > time > frequency Spatio-temporal: 71 %–82 % frequency domain: 53 %–82 % amplitude domain: 68 %–88 % **3. Classification (Acc. Sens. Spec. AUC):** Acc. 53 %–88 %, Sens. 48 %–96 %, Spec.45 %–82 %, AUC 0.56−0.87
[31] Fan et al., 2023, China	**Gait Features extracted (n = 6)** Spatio-temporal: ①large step gait speed (m/s), ②average gait speed (m/s), ③total cadence (step/s), ④large step cadence (step/s), ⑤step length (m), ⑥step time (s) **Other walking features (n = 4)**	***NA***	**Features selection:** Independent t-test or Chi-square test **Features contribution:** SHAP value **Classification:** 5 ML (RF, DT, naïve Bayes, NN, SGD)	**1. Features selection:** variables with the *p < 0.05***2. Features contribution:** large step walking speed > average step size > large step cadence **3. Classification**: only gait features: Acc. 47.49−63.6% only demographic and clinical features: Acc. 52.53−63.5% all features: Acc. 58.42−71.11% selected features: Acc. 51.46−68.74%
[32] Abbas et al., 2022, France	**Gait Features extracted (n = 6) 1. Spatio-temporal:** ①average cadence in 5 sec windows **2. Frequency domain:** ②periodicity of the signal **3. Amplitude domain:** ③25% range of the acceleration magnitude **4. Gait dynamics:** ④variability-time series **5. Others:** ⑤⑥standard deviation and kurtosis of the outcome of AR model	***NA***	**Features selection:** Wilcoxon Rank Sum Test and Kruskal-Wallis H Test (p < 0.05) **Features contribution:** Relief ranking **Classification:** 5 ML (NN, SVM R, KNN, RF, GBM)	**1. Features selection:** see Table 1 **2. Features contribution:** ⑤>③>④>①>②>⑥ **3. Classification (Acc.):** NN: 88.18%; SVMg: **88.5%**; KNN: 86.26%; RF: 87.26%; GBM: 87.51%
[42] Soltani et al., 2021, Switzerland	**Gait speed** average, mode, median, 75th, 90th, 95th percentiles, standard deviation (SD), and maximum of gait speed at each window size (30 s, 30−120 s, >120 s)	***NA***	**Features selection:***NA***Features contribution:** descending order by AUC **Classification:** Logistic regression	**1. Features selection:***NA***2. Features contribution:** 95th percentile got best scores (AUC 0.8) **3. Classification (AUC, AIC, BIC):** Model A: only using duration (AUC 0.763) Model B: Combine with any speed metric (AUC 0.779−0.8)
***Classification model based on gait and DPA parameters (n = 3)***				
[36] Abbas et al., 2023, France	**Gait Features (n = 1) 1. Spatio-temporal:***NA***2. Frequency domain:** ①Periodicity **3. Amplitude domain:***NA***4. Gait dynamics:***NA*	**DPA Features (n = 4) 1. Postural and Transition**: ②steps (24 h) **2. Variability:***NA***3. PA Pattern:** ③activity rate, ④energy expenditure (EE), ⑤sleep efficiency	**Feature selection:** Wilcoxon signed rank test, p < 0.05 **Features contribution:***NA***Classification:** 5 ML (NN, SVM, KNN, RF, GBM)	**1. Feature selection:** ①②④ **2. Features contribution**: *NA***3. Classification:** Model 3 is better Model 1: features extracted from daily prediction (Acc. 84.21 %–93.51 %, Sens. 76.61 %–92.88 %, Spec. 90.91 %–95.27 %) Model 2: features extracted from 5-days (averaging) (Acc. 84.4 %–85.32 %, Sens. 73.68 %–78.95 %, Spec. 92.31 %–96.15 %) Model 3: features extracted from 5-days (voting) (Acc. 94.5 %–97.25 %, Sens. 91.23 %–98.25 %, Spec. 91.23 %–98.25 %)
[37] Park et al., 2021, USA	**Gait Features (n = 1) 1. Spatio-temporal:** ①cadence (1/min) **2. Frequency domain:***NA***3. Amplitude domain:***NA***4. Gait dynamics:***NA*	**DPA Features (n = 11) 1. Postural and Transition**: ②% of lying, ③% of sitting, ④% of standing**, ⑤**% of walking, ⑥steps (24 h), duration of ⑦SiSt & ⑧StSi, ⑨number of ⑨SiSt & ⑩StSi **2. Variability:***NA***3. PA Pattern:** ⑪maximal steps in walking bouts, ⑫average steps of walking bouts	**Features contribution:** ANOVA F-statistic **Feature selection:** ANOVA F-statistic and RFE rank test **Classification:** logistic regression	**1.Feature selection:** p < 0.05 (n = 11 except②), **2.Features contribution:** ①>④>⑤>⑪ **3.Classification: Model 1 (11 features):** AUC 0.79, Sens. 71.8%, Spec. 74.2%, Acc. 73.2% **Model 2 (4 optimal features):** AUC 0.77, Sens. 72.2%, Spec. 70.0%, Acc. 71.3%
[41] Kumar et al., 2020, Mexico	**Gait Features (n = 8) 1. Spatio-temporal:** ①step time **2. Frequency domain:** ②PSD max (W/Hz), ③PSD width (Hz), ④Dominant frequency (Hz) **3. Amplitude domain:***NA***4. Gait dynamics:** ⑤variability-Spatio-temporal, ⑥variability-PSD slope(W), ⑦asymmetry, ⑧irregularity	**DPA Features (n = 6) 1. Postural and Transition:***NA***2. Variability**: ⑨CoV of walking bouts **3. PA Pattern:** ⑩number of continuous walk (48 h), ⑪total continuous walking duration (sec./48 h), ⑫max walking bouts (sec./48 h), ⑬max number of continuous steps(48 h), ⑭% of non-continuous walking duration(48 h)	**Features selection:** A stepwise logistic model **Features contribution:** ANOVA F-statistic **Classification:** Logistic regression	**1. Feature selection:** ④⑤⑬ **2. Features contribution**: ④>⑤>⑬ **3. Classification:** model based on features extracted from 60 s walking bouts better Acc. 77.7%, Sens. 76.8%, Spec. 80%, AUC, 0.84

*NA*, no information was reported; AP, Anteroposterior; ML, mediolateral; VT, vertical; PSD, Power Spectral Density; RMS, Root Mean Square; PA, Daily Physical Activity; CoV, Coefficient of Variation; StSi, Stand to Sit; SiSt, Sit to Stand; MtV, Moderate to Vigorous Physical Activity; ANOVA, Analysis of Variance; MONCOVA, multivariate analysis of covariance; # Cohen’s d calculated manually by mean and standard deviation; N-P, non-frail vs pre-frail; N-F, non-frail vs frail; F-P, frail vs pre-frail; ML, Machine Learning; NN, Neural Network; SVM, Support Vector Machine; R, Regression; KNN, k-Nearest Neighbors; RF, Random Forest; GBM, Gradient Boosting Machine; NB: Naive Bayes; Log Logistic Regression; Acc., Accuracy; Sens., Sensitivity; Spec., Specificity; AUC, Area Under the Curve; AIC, Akaike Information Criterion; BIC, Bayesian Information Criterion; RFE, recursive feature elimination technique.

### 2.5 Data synthesis

Parameters were categorized based on the type of analysis and the information provided. Categories of gait parameters included: 1) Spatio-temporal (stride time, gait speed, step length, cadence, stance time, double support time); (2) Frequency domain (power spectral density analysis, gait periodicity); (3) Amplitude domain (acceleration magnitude and range in the anteroposterior, vertical, and mediolateral directions); (4) Gait dynamics (variability, regularity, symmetry); and (5) Other gait parameters (e.g., ratios). If daily physical activity (DPA) parameters were reported, they were categorized as: (1) Postural and Transition (lying, sitting, standing, sit-to-stand, stand-to-sit, walking); (2) Variability (coefficient of variation); (3) PA pattern (e.g., sleep, moderate physical activity, continuous walking bouts (>60 s)).

*P*-values <0.05 were considered to reflect significant differences between groups, and Cohen’s *d* were used to assess effect sizes, *d*-values >0.5 were considered as sufficient effect size. If Cohen’s *d* was not reported, it was calculated using the reported mean and standard deviation values (Table 2).

For studies that included a classification model based on gait parameters or a combination of gait and DPA parameters, model performance outcomes such as AUC (Area Under the Curve), accuracy, sensitivity, specificity, and feature rankings were extracted (Table 3). As a result, some studies appeared in multiple tables.

## 3 Results

**Fig. 1:**
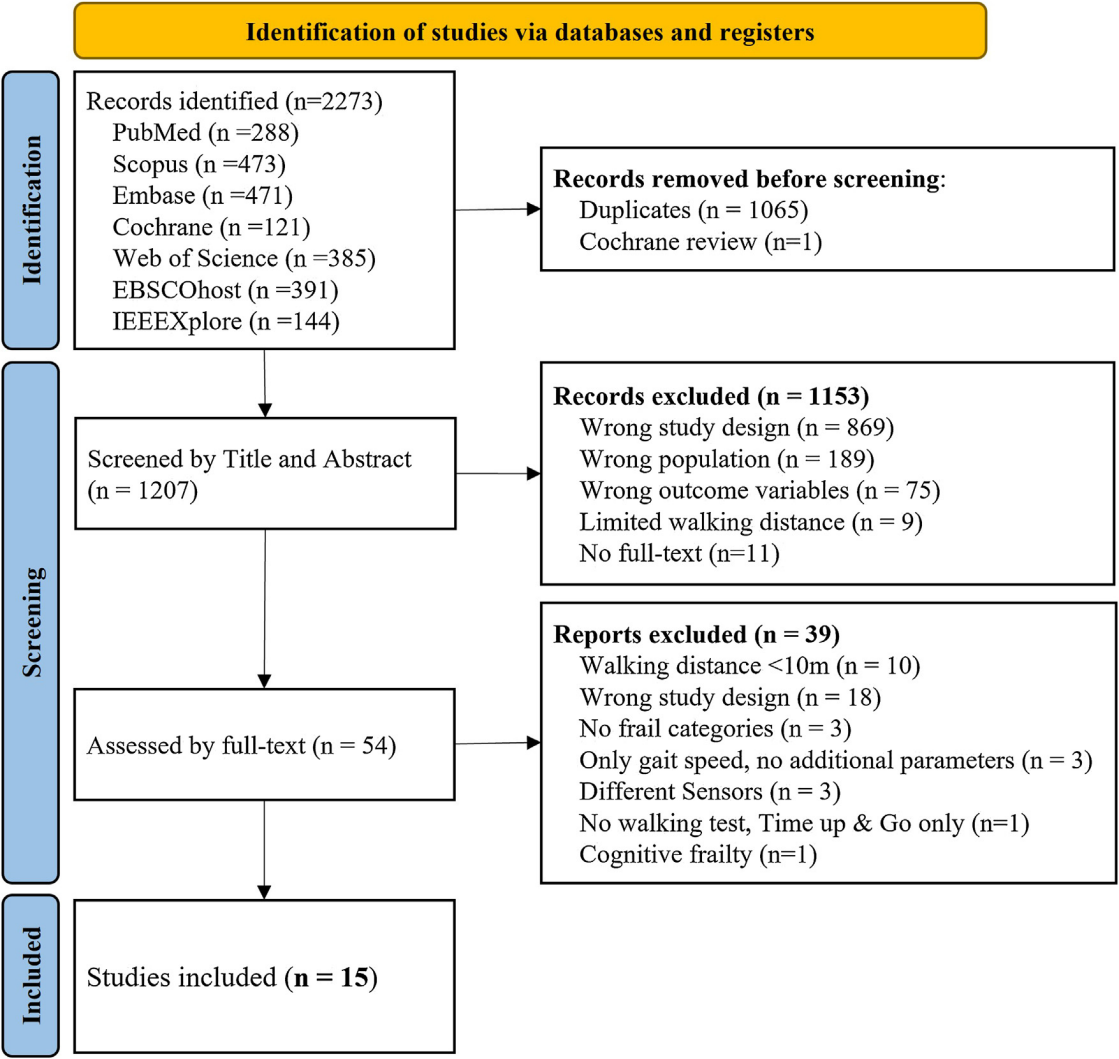
Flow diagram showing paper selection and exclusion criteria.

A total of 2,273 records were identified across seven databases. After removing 1,065 duplicates and excluding 1,154 based on title and abstract, 54 articles remained for full-text screening. Of these, 39 studies were excluded, leaving 15 studies for further review and data extraction [[28], [29], [30], [31], [32], [33], [34], [35], [36], [37], [38], [39], [40], [41], [42]]. A detailed overview of the selection process and exclusion reasons is shown in Fig. 1.

### 3.1 Critical appraisal of evidence

Eleven studies were rated as high quality (12–17 points), while four were rated as moderate quality (7–11 points). All studies scored poorly on the blinding criterion (Item 12). A full summary of the quality assessment is presented in Supplement 1-Table S1.

### 3.2 Characteristics of included studies

Table 1, Table 2, Table 3 provide detailed information on the extracted gait and DPA parameters. All included studies were cross-sectional observational in design. Studies were conducted in the USA (n = 4) [34,35,37,40], Europe (n = 6) [30,32,33,36,38,42], and the remaining five were from China [29,31], Mexico [28,41], and Tanzania [39]. Across these studies, a total of 2,366 participants were involved, with sample sizes ranging from 18 to 1071, and ages spanning from 60 to 92 years. Among the 13 studies that reported gender, with a total sample size of 2,298 participants, 1,718 (75%) were female [[28], [29], [30], [31],[33], [34], [35],[37], [38], [39], [40], [41], [42]]. The other 2 studies included 68 participants with no specification of gender [32,36]. For more details see Table 1.

Walking data were collected in laboratory settings (n = 4) [[28], [29], [30], [31]] and daily life environments (n = 11) [[32], [33], [34], [35], [36], [37], [38], [39], [40], [41], [42]]. The most frequently analyzed gait parameters were from the spatio-temporal (n = 12) [28,29,[31], [32], [33], [34], [35],^37^,^38^,^40^,[33], [34], [35], [36], [37], [38], [39], [40], [41], [42]], followed by gait dynamics (n = 6) [29,32,34,35,39,41], amplitude domain (n = 5) [[28], [29], [30],^32^,^33^], and frequency domain gait parameters (n = 4) [32,34,36,41]. Additional DPA parameters were analyzed in 8 studies [[34], [35], [36], [37], [38], [39], [40], [41]]. Most studies compared non-frail with (pre)frail groups; however, only three studies conducted pairwise comparisons among non-frail, pre-frail, and frail groups using the Fried Phenotype criteria [32,35,40].

In addition, 7 studies tested the performance of a classification model (Table 3), with four focusing solely on gait parameters [[30], [31], [32],^42^] and three incorporating both gait and DPA parameters [36,37,41].

### 3.3 Differences in gait parameters between non-frail and (pre)frail groups

**Fig. 2:**
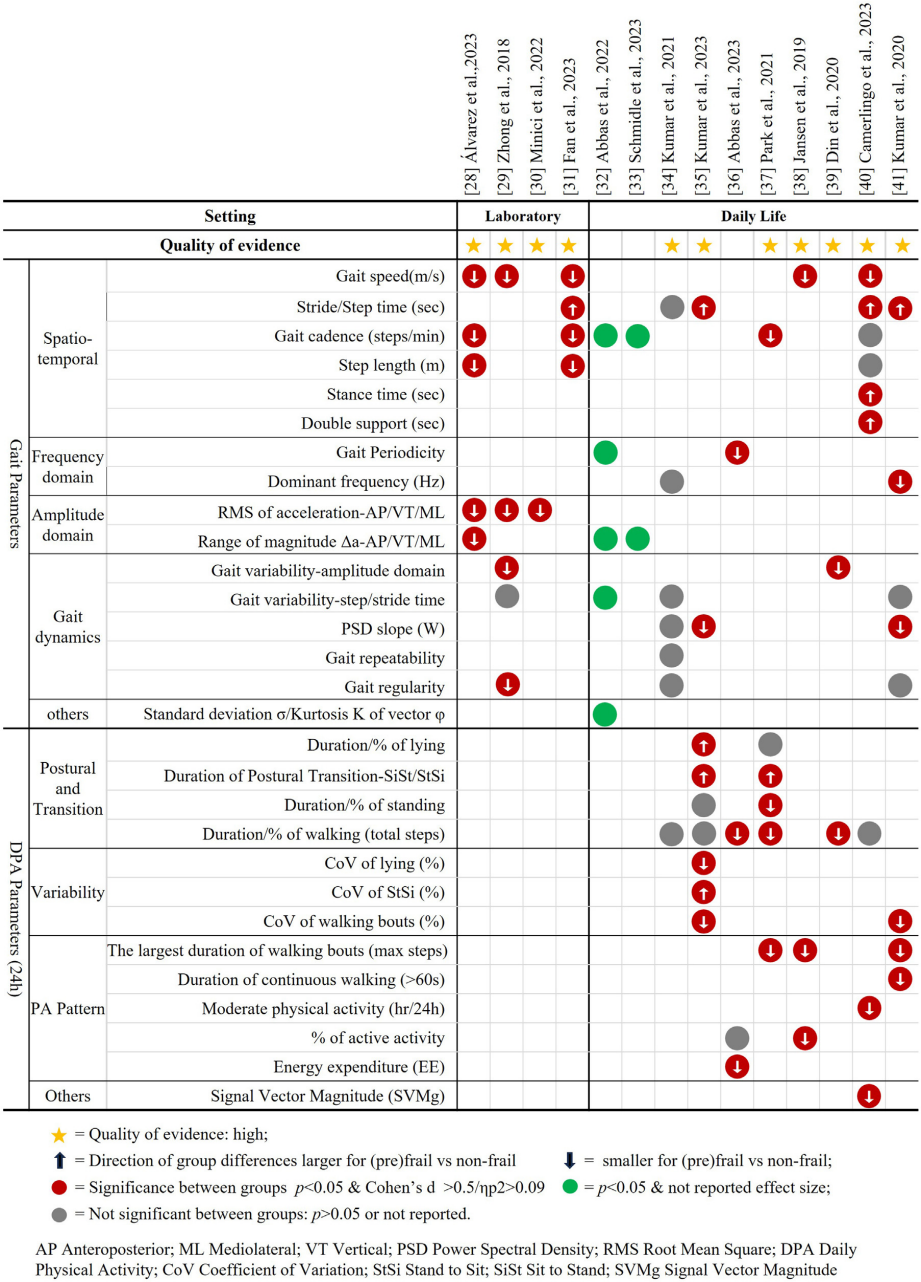
Differences in gait and DPA parameters between non-frail and (pre)frail groups in laboratory (n = 4) and daily life settings (n = 10).

Fig. 2 provides an overview of the differences in gait and DPA parameters between non-frail and (pre)frail groups. As shown, there is substantial variability in the gait parameters assessed across studies, even within specific gait domains. Overall, spatio-temporal parameters consistently showed significant differences between non-frail and (pre)frail groups.

Studies that examined gait parameters beyond the spatio-temporal in controlled laboratory settings [[28], [29], [30], [31]], reported significant differences in amplitude domain parameters [[28], [29], [30]], as well as dynamic domain parameters, such as variability in vertical (VT) magnitude and step regularity, between non-frail and (pre)frail subjects [29], with sufficient effect size. In daily life assessment [[32], [33], [34], [35], [36], [37], [38], [39], [40], [41]], significant differences were observed in both frequency and amplitude domain gait parameters between the two groups [32,33,36,41]. Moreover, dynamic domain gait parameters, including gait variability in VT magnitude [39] and stride time [32], and Power Spectral Density (PSD) slope [35,41], also significantly differed between groups. Certain gait parameters exhibited sufficient effect sizes (d: 0.8–1.49), including the gait periodicity and dominant frequency in the frequency domain [36,41], and dynamic domain metrics such as gait magnitude variability [39] and PSD slope [35,41].

Both in daily life and laboratory assessments, the frail group exhibited an average increase in stride time of 3.25–8.93 % [31,35,40,41], a reduction in gait speed of 0.20 m/s [28,29,31,38,40], a shorter step length by 0.11 m [28,31], and a reduced cadence of 8.22 steps/min [28,31,37] compared to the non-frail group. In the (pre)frail group, all frequency and amplitude domain gait parameters were significantly lower. Specifically, the dominant frequency was reduced by 0.17 Hz [41], the root mean square (RMS) and range of magnitude were [[28], [29], [30]], on average, 25% lower compared to the non-frail group (Table 2).

### 3.4 Differences in DPA parameters between non-frail and (pre)frail groups

As shown in Fig. 2, the duration of walking, postural transitions, and physical activity patterns—including the largest walking bouts [37,38,41], continuous walking bouts (>60 s) [41], and percentage of active activity [38,40]—significantly differed between non-frail and (pre)frail groups, with Cohens *d* > 0.5. The variability of different physical activities also showed significant differences between these groups, including the coefficient of variation (CoV) of lying, stand-to-sit (StSi), and walking bouts [35].

During a 24-h daily routine, (pre)frail subjects showed more inactive patterns. Compared to the non-frail group, the (pre)frail group had shorter walking durations [36,37,39], more frequent but shorter walking bouts (over 50% shorter) [37,38,41], and extended postural transitions such as sit-to-stand (SiSt) [35,37] compared to the non-frail group (Table 2).

### 3.5 Pairwise comparison among pre-frail, frail, and non-frail older adults

Only three studies examined the differences among pre-frail, frail, and non-frail older adults [32,35,40], all of which were conducted in daily life environments. Gait periodicity, range of magnitude, gait variability could effectively distinguish pre-frail individuals from both non-frail and frail participants, with statistically significant differences [32]. Regarding DPA parameters, CoV of walking bouts emerged as the sole metric capable of specifically identifying pre-frail individuals, with significant differences and sufficient effect sizes (d: 0.66, 0.80), showing a 53.73% decrease from non-frailty to pre-frailty and a further 30.87% reduction from pre-frailty to frailty [35] (Table 2, Supplement 2).

### 3.6 Performance of classification models based on sets of parameters

Table 3 presents the findings of studies that reported the performance of frailty classification models, using both logistic regression [37,41,42] and Machine Learning (ML) approaches [[30], [31], [32],^36^]. These frailty classification models were evaluated using metrics such as accuracy, sensitivity, specificity, and Area Under the Curve (AUC). Models that incorporated both gait parameters and DPA outperformed those relying solely on gait parameters [[30], [31], [32],^42^], with the combined models achieving the highest reported accuracy (97.25%), sensitivity (98.25%), and specificity (98.25%) [36,37,41].

Parameters were ranked using various analytical methods, including Relief, SHAP value, regression coefficients, accuracy, and AUC. In the ranking, gait parameters were generally prioritized over DPA parameters, as indicated by ANOVA F-statistic coefficients [37,41]. Among gait parameters, those in the amplitude domain were consistently ranked higher than those in the time and frequency domains [30,32]. Furthermore, the relative rankings of time, frequency, and dynamic domain gait parameters varied across the different models [32,41].

### 3.7 Discussion

The objective of this scoping review was to evaluate the effectiveness of gait parameters, both independently and in combination with Daily Physical Activity (DPA), in distinguishing between pre-frail, frail, and non-frail older adults. We focused specifically on studies utilizing walking distances of at least 10 meters in both controlled and daily life environments. Given the variability in frailty definitions and the heterogeneity in study protocols and outcomes, we grouped outcomes of studies according to gait and DPA parameters and organized the gait parameters into distinct domains. Our review reveals that gait and DPA parameters can effectively differentiate (pre)frail individuals from non-frail older adults.

Consistent with previous reviews including studies with shorter walking distances [[17], [18], [19]], spatio-temporal gait parameters showed significant differences between non-frail and (pre)frail groups across studies. This indicates that prolonged (>10 m) walking assessments yield similar average spatio-temporal gait measurements—including gait speed, step time, and step length—as shorter walks. In controlled environments, amplitude-domain gait parameters also revealed significant differences in frailty status. However, a significant interaction effect was observed between these parameters (RMS of acceleration) and gait speed, emphasizing their close relations. The RMS of acceleration constitutes a statistical measure of the magnitude of acceleration, and correlates with walking speed, that is, subjects will have higher RMS when they walk faster [43,44].

In our current review, we specifically concentrated on studies involving prolonged walking. This approach may improve ecological validity by capturing a broader spectrum of gait patterns and providing a more accurate reflection of functional capacity. Dynamic gait parameters, hypothesized to reflect gait variability in daily life, have been successfully used to differentiate between groups with conditions such as falls, cognitive impairment, and low back pain [15,21,45]. However, relatively few frailty studies have examined dynamic gait parameters, and results are often inconsistent. Variations in factors such as the day of measurement (weekday vs. weekend) and the duration of walking bouts used to extract gait data (ranging from 10 seconds to over 60 seconds) may contribute to these discrepancies, highlighting the need for careful consideration of environmental and methodological influences when interpreting frailty-related gait outcomes.

Considering the inclusion criteria of walking assessment of at least 10 meters, most qualifying studies were conducted in uncontrolled, daily-life settings where DPA were also recorded. Models combining gait and DPA parameters to classify frail and non-frail adults performed better than those using either gait or DPA parameters alone. In daily-life settings, gait parameters derived from longer walking bouts have shown better (pre)frailty assessment performance compared to shorter bouts [42]. This presumably relates to the higher likelihood of abnormal gait occurring in more challenging situations, such as fatigue during DPA, particularly in older adults with impaired walking ability. In addition, laboratory assessments may be affected by the Hawthorne effect—where participants modify their walking behavior when they know they're being observed. Studies have shown that gait parameters can differ between unsupervised and supervised settings (e.g., in laboratories or hospitals) [46,47]. Therefore, unsupervised prolonged walking assessment and/or long-term monitoring (hours or days) likely provides a more accurate representation of typical daily walking patterns.

Identifying pre-frailty is critical for early intervention, potentially preventing or delaying the progression to frailty [48,49]. However, only three study attempted to classify all three frailty stages—non-frail, pre-frail, and frail. Preliminary results indicate that pre-frailty can be identified using a combination of gait parameters and DPA variability in walking, although further validation is required. The initial findings also suggest that incorporating DPA measures can enhance the accuracy of frailty classification models.

However, the included studies were cross-sectional, which limits our ability to evaluate the predictive power of gait and DPA parameters for pre-frailty and frailty classification over time. Another limitation is the gender imbalance across studies, which may introduce bias. Significant differences in gait patterns, physical activity levels, and frailty prevalence between males and females have been reported [[50], [51], [52]], and future research should account for these variations to ensure more accurate and generalizable results. Moreover, participants were recruited from different environments (e.g., hospital, general community center, nursing house), even in the same study, increasing the bias.

Future research should aim to better understand the distinct gait characteristics of pre-frail individuals and develop robust models that can accurately distinguish between non-frail, pre-frail, and frail states. Regular, long-term monitoring of daily activity patterns could provide valuable insights, although uncontrolled real-world settings may pose challenges in maintaining data quality. Standardizing data processing methods is crucial to improving the comparability of findings. Advanced ML techniques offer promising avenues for improving classification accuracy. This review highlights the need for a standardized pipeline for gait as well as for DPA monitoring and analysis. Standardization should begin with data collection, including participants recruitment, sensor requirements (e.g., sample frequency, number of sensors, localization) and measurement protocols (e.g., walking distance and/or number of measurement days, sample size). A data preprocessing pipeline should be reported and need to specify data span, filtering methods, window size, number of steps included for data analysis. For DPA classification models detailed parameter calculations and performance metrics (e.g., F1-statistics, confusion matrix and/or sensitivity specificity, variable importance outcomes) should be reported in addition to accuracy. While complete standardization may be challenging, and different research questions require varying protocols and methods, implementing a standardized pipeline for data preprocessing with open-source algorithms and thorough reporting would enable better comparison of results and impact analysis. Furthermore, longitudinal studies could provide critical insights into how gait and DPA patterns evolve over time and their predictive value for frailty progression. Such an approach would deepen our understanding of frailty dynamics and improve early detection and intervention strategies.

## Conclusions and implications

This scoping review highlights a critical gap in frailty screening—namely, the challenge of effectively distinguishing between non-frail, pre-frail, and frail individuals. The diversity of methods, protocols, and gait parameters across studies has made it difficult to establish consistent associations between specific gait characteristics and frailty. However, combining gait parameters with DPA data has been shown to improve the accuracy of frailty classification and enhance the ability to categorize individuals within the three frailty stages.

We recommend the implementation of long-term, continuous monitoring using discreet wearable sensors, alongside the standardization of data processing for large-scale acceleration datasets. Such approaches could facilitate earlier frailty detection and support clinical decision-making, and contribute to the development of personalized interventions. Ultimately, this would improve clinical management and enhance the quality of life for older adults.

## CRediT authorship contribution statement

Conceptualization and methodology: Zhang X, Lamoth CJC.

Data curation: Zhang X, Lamoth CJC.

Formal analysis and interpretation of data: all authors.

Drafting of the manuscript (writing and visualization): Zhang X, Lamoth CJC.

Critical revision of the manuscript for important intellectual content: all authors.

## Funding sources

This work was supported by the China Scholarship Council- University of Groningen Scholarship [Grant No.202206170103].

## Declaration of competing interest

The authors declared no potential conflict of interest with respect to the research, authorship, and/or publication of this article.
