# Supplementary material for: Gait parameters and daily physical activity for distinguishing pre-frail, frail, and non-frail older adults: A scoping review
Source: J Nutr Health Aging. 2025 May 14;29(7):100580. doi: 10.1016/j.jnha.2025.100580 (PMC12173000; doi:10.1016/j.jnha.2025.100580)
Supplement: Supplementary file 1 [file mmc1.doc]

**Supplementary material Legends**

**S1** Include and exclude criteria

**S2** Search Strategy

**Table S1** Modified Downs and Black checklist

**S1 Include and exclude criteria**

***INCLUDE***

- Study purpose: for frailty screening/distinguishing/assessment
- Population: healthy and frail/pre-frail older adults
- Methods: gait parameters collected by inertial sensors and walking distance > 10m
- Results: report on the association between frailty and gait parameters

***EXCLUDE***

- Irrelevant: not conform to purpose, population, methods, and results
- Frailty screen used other markers (biomarker/digital marker)
- Gait parameters to screen other diseases (cardiovascular)/fall/mortality/hospital admission
- Study design: Qualitative research; proposal; editorial; intervention study
- Wrong population: patients with stroke, Alzheimer’s disease, and other central nervous system diseases such as Parkinson’s disease or multiple sclerosis
- No frail group: just the difference between age groups or trajectory of gait variables
- Only for system/model/algorithm/technology: just test the accuracy/acceptability/feasibility (sample size <10)
- Without technology/sensors
- No extra gait parameters other than mean gait speed
- Not English

**S2 Search Strategy**

- ***PubMed***

("Frail Elderly"[Mesh] OR "Frailty"[Mesh] OR frail*[tiab])

AND

(“walking” [Mesh] OR gait*[tiab] OR walk*[tiab])

AND

(Inertial Measurement unit*[tiab] OR IMU [tiab] OR sensor*[Title/Abstract] OR technol*[Title/Abstract] OR accelerometer*[Title/Abstract] OR smart watch*[Title/Abstract] OR activity watch*[Title/Abstract] OR "Fitness Trackers "[Mesh] OR fitness tracker*[tiab] OR activity tracker*[tiab])

AND

("Aged"[Mesh] OR "Aging"[Mesh] OR "Age Factors"[Mesh] OR elderly[tiab] OR older patient*[tiab] OR old patient*[tiab] OR older person*[tiab] OR old person*[tiab] OR older subject*[tiab] OR older adult*[tiab] OR old adult*[tiab] OR older people [tiab] OR senior*[tiab] OR very old[tiab] OR geriatr*[tiab] OR very-old[tiab] OR oldest[tiab] OR nonagenarian*[tiab] OR octogenarian*[tiab] OR centenarian[tiab] OR 80-and-older[tiab] OR over-80[tiab] OR over-85[tiab] OR over-90[tiab])

- ***Web of Science***

(TS=(frail*))

AND

(TS=(gait*) OR TS=(walk*))

AND

(TS=(Inertial Measurement unit*) OR TS=(IMU) OR TS=(sensor*) OR TS=(technol*) OR TS=(accelerometer*) OR TS=(smart watch*) OR TS=(activity watch*) OR TS=(Fitness Tracker*) OR TS=(activity tracker*))

AND

(TS=(aged) OR TS=(aging) OR TS=(age factors) OR TS=(elderly) OR TS=(older patient*) OR TS=(old patient*) OR TS=(older person*) OR TS=(old person*) OR TS=(older subject*) OR TS=(older adult*) OR TS=(old adult*) OR TS=(older people) OR TS=(senior*) OR TS=(very old) OR TS=(geriatr*) OR TS=(very-old) OR TS=(oldest) OR TS=(nonagenarian*) OR TS=(octogenarian*) OR TS=(centenarian) OR TS=(80-and-older) OR TS=(over-80) OR TS=(over-85) OR TS=(over-90))

- ***Scopus***

(TITLE-ABS-KEY (frail*))

AND

(TITLE-ABS-KEY (gait*) OR TITLE-ABS-KEY (walk*))

AND

(TITLE-ABS-KEY (“Inertial Measurement unit*”) OR TITLE-ABS-KEY(IMU) OR TITLE-ABS-KEY (sensor*) OR TITLE-ABS-KEY(technol*) OR TITLE-ABS-KEY(accelerometer*) OR TITLE-ABS-KEY(“smart watch*”) OR TITLE-ABS-KEY(“activity watch*”) OR TITLE-ABS-KEY(“Fitness Tracker*”) OR TITLE-ABS-KEY(“activity tracker*”))

AND

(TITLE-ABS-KEY(aged) OR TITLE-ABS-KEY(aging) OR TITLE-ABS-KEY(“age factors”) OR TITLE-ABS-KEY(elderly) OR TITLE-ABS-KEY(“older patient*”) OR TITLE-ABS-KEY(“old patient*”) OR TITLE-ABS-KEY(“older person*”) OR TITLE-ABS-KEY(“old person*”) OR TITLE-ABS-KEY(“older subject*”) OR TITLE-ABS-KEY(“older adult*”) OR TITLE-ABS-KEY(“old adult*”) OR TITLE-ABS-KEY(“older people”) OR TITLE-ABS-KEY(senior*) OR TITLE-ABS-KEY(“very old”) OR TITLE-ABS-KEY(geriatr*) OR TITLE-ABS-KEY(very-old) OR TITLE-ABS-KEY(oldest) OR TITLE-ABS-KEY(nonagenarian*) OR TITLE-ABS-KEY(octogenarian*) OR TITLE-ABS-KEY(centenarian) OR TITLE-ABS-KEY(80-and-older) OR TITLE-ABS-KEY(over-80) OR TITLE-ABS-KEY(over-85) OR TITLE-ABS-KEY(over-90))

- ***Cochrance***

#1 Frail Elderly [MESH]

#2 Frailty

#3 (frail*): ti,ab,kw

#4 #1 or #2 or #3
#5 walking

#6 (gait*): ti,ab,kw OR (walk*):ti,ab,kw

#7 #5 or #6

#8 Fitness Trackers

#9 (“Inertial Measurement unit*”):ti,ab,kw OR (IMU):ti,ab,kw OR (sensor*):ti,ab,kw OR (technol*):ti,ab,kw OR (accelerometer*):ti,ab,kw OR (“smart watch*”):ti,ab,kw OR (“activity watch*”):ti,ab,kw OR (“Fitness Tracker*”):ti,ab,kw OR (“activity tracker*”):ti,ab,kw

#10 #8 or #9

#11 Aged

#12 Aging

#13 Age Factors

#14 (aged):ti,ab,kw OR (aging):ti,ab,kw OR (“age factors”):ti,ab,kw OR (elderly):ti,ab,kw OR (“older patient*”):ti,ab,kw OR (“old patient*”):ti,ab,kw OR (“older person*”):ti,ab,kw OR (“old person*”):ti,ab,kw OR (“older subject*”):ti,ab,kw OR (“older adult*”):ti,ab,kw OR (“old adult*”):ti,ab,kw OR (“older people”):ti,ab,kw OR (senior*):ti,ab,kw OR (“very old”):ti,ab,kw OR (geriatr*):ti,ab,kw OR (very-old):ti,ab,kw OR (oldest):ti,ab,kw OR (nonagenarian*):ti,ab,kw OR (octogenarian*):ti,ab,kw OR (centenarian):ti,ab,kw OR (“80-and-older”):ti,ab,kw OR (“over-80”):ti,ab,kw OR (“over-85”):ti,ab,kw OR (“over-90”):ti,ab,kw

#15 #11 or #12 or #13 or #14

#16 #4 and #7 and #10 and #15

- ***Ebscohost***

#1 frail*

#2 gait* OR walk*

#3 “Inertial Measurement unit*” OR IMU OR sensor* OR technol* OR accelerometer* OR “smart watch*” OR “activity watch*” OR “Fitness Tracker*” OR “activity tracker*”

#4 aged OR aging OR “age factors” OR elderly OR “older patient*” OR “old patient*” OR “older person*” OR “old person*” OR “older subject*” OR “older adult*” OR “old adult*” OR “older people” OR senior* OR “very old” OR geriatr* OR very-old OR oldest OR nonagenarian* OR octogenarian* OR centenarian OR 80-and-older OR over-80 OR over-85 OR over-90

- ***Embase***

('frailty'/exp OR 'frail elderly'/exp OR frail*:ti,ab,kw)

AND

('walking'/exp OR 'walking parameters'/exp OR gait*:ti,ab,kw OR walk*:ti,ab,kw)

AND

('wearable sensor'/exp OR 'inertial sensor'/exp OR 'accelerometer'/exp OR 'smart watch'/exp OR ‘Inertial Measurement unit*’:ti,ab,kw OR IMU:ti,ab,kw OR sensor*:ti,ab,kw OR technol*:ti,ab,kw OR accelerometer*:ti,ab,kw OR ‘smart watch*’:ti,ab,kw OR ‘activity watch*’:ti,ab,kw OR ‘Fitness Tracker*’:ti,ab,kw OR ‘activity tracker*’:ti,ab,kw)

AND

('aged'/exp OR aged:ti,ab,kw OR aging:ti,ab,kw OR ‘age factors’:ti,ab,kw OR elderly:ti,ab,kw OR ‘older patient*’:ti,ab,kw OR ‘old patient*’:ti,ab,kw OR ‘older person*’:ti,ab,kw OR ‘old person*’:ti,ab,kw OR ‘older subject*’:ti,ab,kw OR ‘older adult*’:ti,ab,kw OR ‘old adult*’:ti,ab,kw OR ‘older people’:ti,ab,kw OR senior*:ti,ab,kw OR ‘very old’:ti,ab,kw OR geriatr*:ti,ab,kw OR very-old:ti,ab,kw OR oldest:ti,ab,kw OR nonagenarian*:ti,ab,kw OR octogenarian*:ti,ab,kw OR centenarian:ti,ab,kw OR ‘80-and-older’:ti,ab,kw OR ‘over-80’:ti,ab,kw OR ‘over-85’:ti,ab,kw OR ‘over-90’:ti,ab,kw)

- ***IEEEXplore***

("All Metadata":frail* AND ("All Metadata":gait* OR walk*) AND ("All Metadata":“Inertial Measurement unit*” OR "All Metadata":IMU OR "All Metadata":sensor* OR "All Metadata":technol* OR "All Metadata":accelerometer* OR "All Metadata":“activity tracker*”))

**Table S1** Modified Black and Downs Checklist

| **Items/Studies** | [28]  Álvarez et al.,  2023 | [29]  Zhong  et al.,  2018 | [30]  Minici  et al.,  2022 | [31]  Fan  et al.,  2023 | [32]  Abbas  et al.,  2022 | [33]  Soltani  et al.,  2021 | [34]  Kumar  et al.,  2021 | [35]  Kumar  et al.,  2023 | [36]  Abbas  et al.,  2023 | [37]  Park  et al.,  2021 | [38]  Jansen  et al.,  2019 | [39]  Din  et al.,  2020 | [40]  Camerlingo  et al.,  2023 | [41]  Kumar  et al.,  2020 | [42]  Schmidle  et al.，  2023 |
| --- | --- | --- | --- | --- | --- | --- | --- | --- | --- | --- | --- | --- | --- | --- | --- |
| ***REPORTING*** |  |  |  |  |  |  |  |  |  |  |  |  |  |  |  |
| 1. Is the objective of the study clear? | 1 | 1 | 1 | 1 | 1 | 1 | 1 | 1 | 1 | 1 | 1 | 1 | 1 | 1 | 1 |
| 2. Are the main outcomes clearly described in the Introduction or Methods? | 1 | 1 | 1 | 1 | 1 | 1 | 1 | 1 | 1 | 1 | 1 | 1 | 1 | 1 | 1 |
| 3. Are characteristics of the patients included in the study clearly described? | 1 | 1 | 1 | 1 | 0 | 0 | 1 | 1 | 0 | 1 | 1 | 1 | 1 | 1 | 1 |
| 4. Are the interventions clearly described? | 1 | 1 | 1 | 1 | 1 | 1 | 1 | 1 | 1 | 0 | 1 | 1 | 1 | 1 | 1 |
| 5. Are the distributions of principal confounders in each group of subjects clearly described? | 1 | 1 | 1 | 1 | 0 | 0 | 1 | 1 | 0 | 1 | 1 | 1 | 1 | 1 | 0 |
| 6. Are the main findings of the study clearly described? | 1 | 1 | 1 | 1 | 1 | 1 | 1 | 1 | 1 | 1 | 1 | 1 | 1 | 1 | 1 |
| 7. Does the study estimate random variability in data for main outcomes? | 1 | 0 | 1 | 1 | 1 | 0 | 1 | 1 | 1 | 1 | 1 | 1 | 1 | 1 | 1 |
| 8. Have actual probability values been reported for the main outcomes except probability < 0.001? | 0 | 1 | 1 | 1 | 0 | 0 | 1 | 1 | 1 | 1 | 1 | 1 | 1 | 1 | 1 |
| 9. Is the source of funding clearly stated? | 1 | 1 | 1 | 1 | 1 | 0 | 1 | 1 | 1 | 1 | 1 | 1 | 1 | 1 | 1 |
| ***EXTERNAL VALIDITY*** |  |  |  |  |  |  |  |  |  |  |  |  |  |  |  |
| 10. Were subjects who were asked to participate in the study representative of the entire population recruited? | 1 | 1 | 1 | 1 | 0 | 1 | 1 | 1 | 0 | 1 | 1 | 1 | 0 | 1 | 1 |
| 11.Were those subjects who were prepared to participate representative of the recruited population? | 1 | 1 | 1 | 1 | 0 | 1 | 1 | 0 | 0 | 1 | 0 | 0 | 1 | 1 | 1 |
| ***INTERNAL VALIDITY*** |  |  |  |  |  |  |  |  |  |  |  |  |  |  |  |
| 12. Was an attempt made to blind those measuring the main outcomes? | 0 | 0 | 0 | 0 | 0 | 0 | 0 | 0 | 0 | 0 | 0 | 0 | 0 | 0 | 0 |
| 13. If any of the results of the study were based on data dredging, was this made clear? | 1 | 1 | 1 | 1 | 1 | 1 | 1 | 1 | 1 | 1 | 1 | 1 | 1 | 1 | 1 |
| 14. Were the statistical tests used to assess main outcomes appropriate? | 1 | 1 | 1 | 1 | 1 | 1 | 1 | 1 | 1 | 1 | 1 | 1 | 1 | 1 | 1 |
| 15. Were main outcome measures used accurate? (valid and reliable) | 1 | 1 | 1 | 1 | 1 | 1 | 1 | 1 | 1 | 1 | 1 | 1 | 1 | 1 | 1 |
| ***INTERNAL VALIDITY-CONFOUNDING (SELECTION BIAS)*** |  |  |  |  |  |  |  |  |  |  |  |  |  |  |  |
| 16. Was there adequate adjustment for confounding in the analysis from which main findings were drawn? | 1 | 1 | 1 | 1 | 0 | 1 | 1 | 1 | 0 | 1 | 0 | 1 | 1 | 1 | 1 |

| **Items/Studies** | | | | | [28]  Álvarez et al.,  2023 | [29]  Zhong  et al.,  2018 | [30]  Minici  et al.,  2022 | [31]  Fan  et al.,  2023 | [32]  Abbas  et al.,  2022 | [33]  Soltani  et al.,  2021 | [34]  Kumar  et al.,  2021 | [35]  Kumar  et al.,  2023 | [36]  Abbas  et al.,  2023 | [37]  Park  et al.,  2021 | [38]  Jansen  et al.,  2019 | [39]  Din  et al.,  2020 | [40]  Camerlingo  et al.,  2023 | [41]  Kumar  et al.,  2020 | [42]  Schmidle  et al.，  2023 |
| --- | --- | --- | --- | --- | --- | --- | --- | --- | --- | --- | --- | --- | --- | --- | --- | --- | --- | --- | --- |
| ***POWER*** | | | | |  |  |  |  |  |  |  |  |  |  |  |  |  |  |  |
| 17. Was the study sufficiently powered to detect clinically important effects where probability value for a difference due to chance is < 5%? | | | | | 1 | 1 | 1 | 1 | 1 | 1 | 1 | 1 | 1 | 1 | 1 | 1 | 1 | 1 | 1 |
| **Total score** | | | | | **15** | **15** | **16** | **16** | **10** | **11** | **16** | **15** | **11** | **15** | **14** | **15** | **15** | **16** | **15** |
| **Risk of bias** | | | | | low | low | low | low | moderate | moderate | low | low | moderate | low | low | low | low | low | moderate |
|  |  |  |  |  | | | | | | | | | | | | | | | |
