# Supplementary material for: Gait parameters and daily physical activity for distinguishing pre-frail, frail, and non-frail older adults: A scoping review
Source: J Nutr Health Aging. 2025 May 14;29(7):100580. doi: 10.1016/j.jnha.2025.100580 (PMC12173000; doi:10.1016/j.jnha.2025.100580)
Supplement: Supplementary file 2 [file mmc2.pptx]

## Slide 1
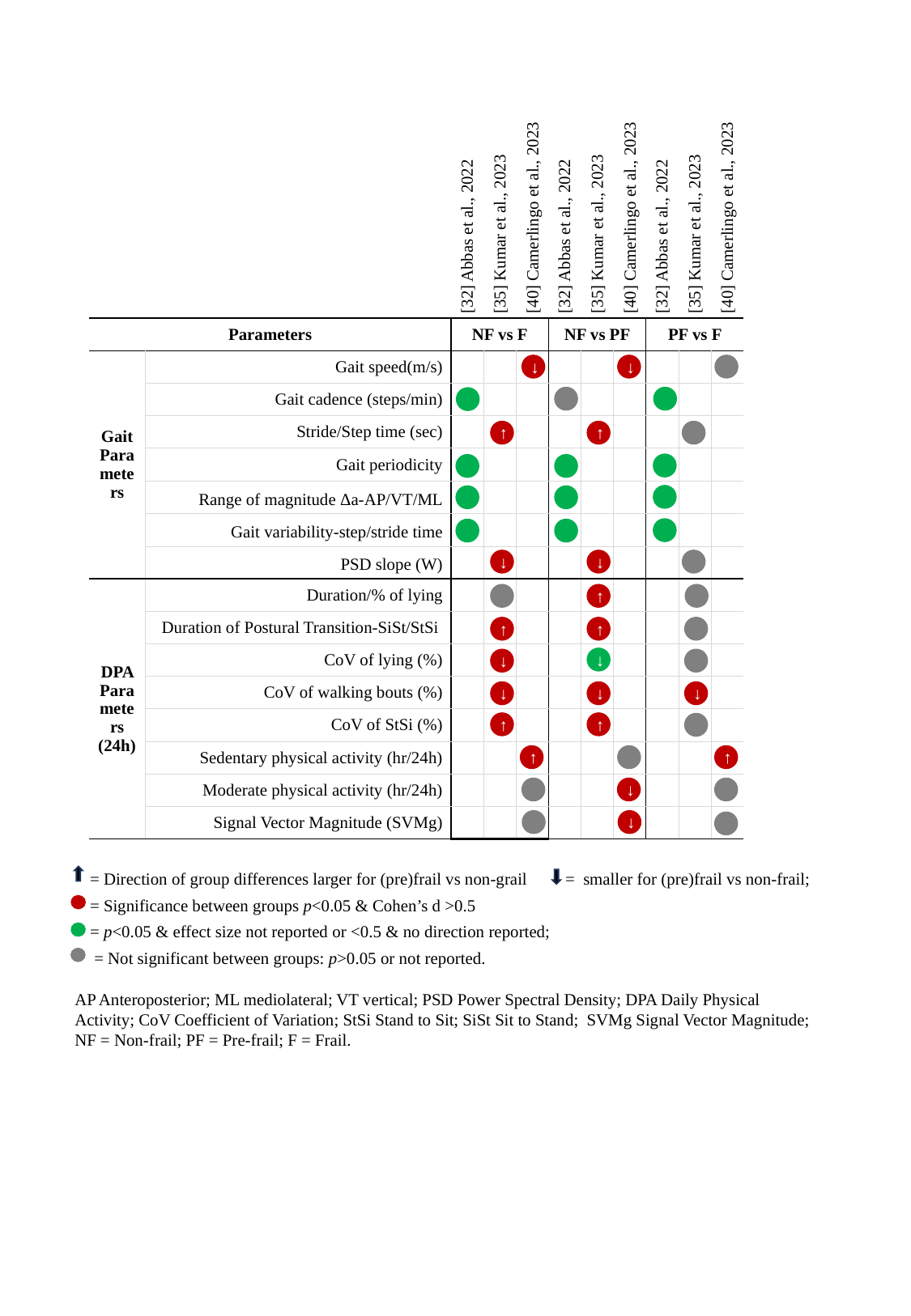

[32] Abbas et al., 2022
[35] Kumar et al., 2023
[40] Camerlingo et al., 2023
[32] Abbas et al., 2022
[35] Kumar et al., 2023
[40] Camerlingo et al., 2023
[32] Abbas et al., 2022
[35] Kumar et al., 2023
[40] Camerlingo et al., 2023
| Parameters | | NF vs F | | | NF vs PF | | | PF vs F | | |
| --- | --- | --- | --- | --- | --- | --- | --- | --- | --- | --- |
| Gait Parameters | Gait speed(m/s) | | | | | | | | | |
| | Gait cadence (steps/min) | | | | | | | | | |
| | Stride/Step time (sec) | | | | | | | | | |
| | Gait periodicity | | | | | | | | | |
| | Range of magnitude Δa-AP/VT/ML | | | | | | | | | |
| | Gait variability-step/stride time | | | | | | | | | |
| | PSD slope (W) | | | | | | | | | |
| DPA Parameters (24h) | Duration/% of lying | | | | | | | | | |
| | Duration of Postural Transition-SiSt/StSi | | | | | | | | | |
| | CoV of lying (%) | | | | | | | | | |
| | CoV of walking bouts (%) | | | | | | | | | |
| | CoV of StSi (%) | | | | | | | | | |
| | Sedentary physical activity (hr/24h) | | | | | | | | | |
| | Moderate physical activity (hr/24h) | | | | | | | | | |
| | Signal Vector Magnitude (SVMg) | | | | | | | | | |
↓
↓
↑
↑
↓
↓
↑
↑
↑
↓
↓
↓
↓
↓
↑
↑
↑
↑
↓
↓
= Direction of group differences larger for (pre)frail vs non-grail = smaller for (pre)frail vs non-frail;
= Significance between groups p<0.05 & Cohen’s d >0.5
= p<0.05 & effect size not reported or <0.5 & no direction reported;
 = Not significant between groups: p>0.05 or not reported.
AP Anteroposterior; ML mediolateral; VT vertical; PSD Power Spectral Density; DPA Daily Physical Activity; CoV Coefficient of Variation; StSi Stand to Sit; SiSt Sit to Stand; SVMg Signal Vector Magnitude; NF = Non-frail; PF = Pre-frail; F = Frail.
